# Profiling Chinese EFL students’ technology-based self-regulated English learning strategies

**DOI:** 10.1371/journal.pone.0240094

**Published:** 2020-10-29

**Authors:** Zhujun An, Zhengdong Gan, Chuang Wang

**Affiliations:** Faculty of Education, University of Macau, Macao, China; Lingnan University, HONG KONG

## Abstract

This article reports the development and validation of an instrument, the Technology-Based Self-Regulated English Learning Strategies Scale (TSELSS), in terms of its multifaceted structure of self-directed use of technology in English learning among Chinese university EFL students. TSELSS was developed through a three-phase process, focusing on the domain of self-regulated English learning in technology-assisted conditions. The first phase involved the generation of an item pool, the second a pilot study (*N* = 164) aimed at identifying the factor structure of TSELSS using exploratory factor analysis, and the third an examination of the psychometric properties of the revised TSELSS using confirmatory factor analysis with another independent sample of students (*N* = 525). Furthermore, the concurrent validity of TSELSS was investigated through correlations with students’ English language self-efficacy and English learning outcomes. The final version of the scale is made up of five types of technology-based self-regulated English learning strategies: *motivational regulation strategies*, *goal setting and learning evaluation*, *social strategies*, *technology-based English song and movie learning*, and *technology-based vocabulary learning*. The TSELSS can be used as an evaluation tool to appraise EFL students’ technology-based self-regulated English learning experience, and as a research tool to investigate more associations between technology-based self-regulated strategic English learning and other contextual and learner individual factors.

## 1. Introduction

Technology-enhanced language learning has been widely acclaimed for its various facets of the power of technology for language learning in which, with the assistance of technology, learners are provided with diversified learning opportunities [1, 2]. Indeed, technology not only constitutes an important learning space by providing learners with flexible learning venues across time [3] but also impacts on their language learning motivation and learning outcomes [4, 5]. While technology environments have potential to be powerful learning tools for fostering students’ learning, learning in such an environment requires learners to regulate their learning, that is, to make decisions about what to learn, how to learn it, when to modify plans and strategies, and when to increase effort [6, 7]. In other words, self-regulation becomes a vital factor for effective learning in technology-based learning context [8, 9]. Although second language acquisition researchers have shown an increasing interest in the adoption of self-regulated learning strategies and their correlation with other individual variables [10–12], current language learner strategy studies have not given enough attention to learners’ strategic learning in technology-using conditions [13]. Understanding EFL learners’ technology-based self-regulated English learning experience is important because this informs educators on how they can support students to create a more effective learning environment across time and space. However, there has been a lack of systematic and theory-driven inquiry into learners’ self-regulated technology-based language learning experience as current research has largely focused on effectiveness of technology applications in the language classroom [9, 14]. A possible explanation is that there is a lack of valid and theory-driven instruments for investigating learners’ self-regulated language learning in technology-using conditions.

The lack of valid and theory-driven instruments for measuring strategic language learning in technology-using settings apparently relates to little consensus in the literature about how to conceptualise and measure technology-based self-regulated learning in the second language learning context. For example, Barnard and her colleagues [8] developed the Online Self-regulated Learning Questionnaire (OSLQ) to assess students’ self-regulated learning in online and blended learning environments. The online self-regulated learning constructs operationalized in OSLQ include: 1) environment structuring; 2) goal setting; 3) time management; 4) help seeking; 5) task strategies; and 6) self-evaluation. In Lai and Gu’s [9] survey of students’ self-regulated use of information and communication technologies in their language learning, however, technology-mediated self-regulated language learning experience was measured by the following subscales: 1) goal commitment regulation; 2) resource regulation; 3) affection regulation; 4) culture learning regulation; 5) metacognition regulation; and 6) social connection regulation. Also in the field of second language acquisition, the most widely used instrument to measure learners’ learning strategies is the Strategy Inventory for Language Learning (SILL) developed by Oxford [15], which contains six types of language learning strategies (e.g. memory, cognitive, metacognitive, social strategies, etc.). While the validity of SILL has been established in previous learner strategy research, SILL obviously does not capture patterns of learning strategies that are idiosyncratic to technology-using conditions particularly in an EFL context where classroom opportunities for interaction are usually limited and technology-assisted learning is found to enhance language learning outcomes in self-regulated learners.

Clearly, without a valid and theory-driven instrument needed to evaluate university EFL students’ technology-based self-regulated English learning strategies, our knowledge of the extent to which students engage in technology-based English learning and how this type of language learning contributes to their English learning achievement will be limited. Consequently classroom intervention procedures will not be as effective as possible. Against this backdrop, the current study attempts to develop and validate an instrument, the Technology-Based Self-Regulated English Learning Strategies Scale (TSELSS), in terms of its multifaceted structure of self-directed use of technology in English learning among Chinese university EFL students. The development and validation of the TSELSS was grounded in the self-regulated learning (SRL) theory and Koole’s framework for the rational analysis of mobile education (i.e., FRAME). The paper also explores how EFL students’ technology-based self-regulated learning experience is associated with their English language self-efficacy and English learning outcomes.

## 2. Theoretical framework

### 2.1 Self-regulated learning

SRL refers to self-generated thoughts, feelings, and behaviors that are oriented to attaining individuals’ personal goals [16]. In other words, self-regulated learning concerns how students become masters of their own learning processes [17]. As suggested by many researchers [18–21], self-regulation is a dynamic process involving cognitive, affective, motivational, and behavioral components that provide learners with the capacity to adjust his or her actions to achieve particular goals in changing educational settings. As such, learners purposefully activate, sustain, and adjust their cognitions, affects, and actions to achieve their learning goals by employing different strategies [22]. Self-regulated learners are believed to be able to set their learning goals, establish a more productive environment, monitor their understanding and modify their plans, strategies, and effort in relation to changing contextual conditions [22]. Significantly, over the past two decades, general SRL conceptions have somewhat changed, and there has been a shift from the conceptualization of SRL as a relatively stable individual inclination to respond to a range of learning situations in a typical way [18]. Consequently, domain and situational specificity were brought to the foreground, and there has been a growing interest in the dynamic process of SRL in different learning environments. For instance, researchers are interested to investigate learners’ use of technology to self-regulate their language learning [9]. Meanwhile, influenced by socio-cognitive models of learning, current understanding of self-regulation of learning has also evolved from an emphasis on meta-cognition into a recognition of its multidimensional nature including, for example, the regulation of motivational factors that affect learning [23, 24]. This evolution in understanding of self-regulation is best illustrated in Pintrich’s [24] own comment on the development of the MSLQ [25, 26] “which does not include any measures of students’ attempts to monitor, control, and regulate their motivation or affect, making it a limited instrument in terms of assessing important motivational or affective self-regulatory strategies”(p.397).

As outlined above, determining level of self-regulation usually involves the process of assessing how well students have developed the inclusive array of learning strategies that are typically classified in terms of cognition, metacognition, social behavior, and motivational regulation [27, 28]. Specifically, cognitive strategies are like construction workers, with which learners put together, consolidate, elaborate, and transform knowledge of the language and culture [27]. Metacognitive strategies, however, are like construction manager, aiding the learners in focusing, planning, obtaining resources, organizing, coordinating, monitoring, and evaluating the construction of L2 knowledge [27]. Social behavioral strategies describe individuals’ attempt to control their learning behavior under the influence of contextual and environmental factors, such as seeking social assistance through the Internet [29], while motivational regulation strategies are described as the various actions or tactics that students use to maintain or increase their effort or persistence at a particular academic task [30, 31]. Early self-regulation researchers documented positive relationship between the use of SRL strategies and student performance on standardized tests [32]. Subsequent research also showed that effective learners better regulate their learning by activating, deploying, and modifying cognitive, metacognitive, and behavioral processes prior to, during, and following learning [20, 33].

In the field of second language acquisition, there is a consensus among researchers and practitioners that language learners can achieve greater success in their language learning and use if they are more strategic in their efforts. Language learning strategies have been found to vary in relation to cultures and situations. For example, Anam and Stracke [34] found that Indonesian school students used cognitive strategies at moderate frequency but socio-affective strategies and metacognitive strategies at high frequency, and that students who possessed a higher sense of English efficacy tended to use cognitive, socio-affective, and metacognitive strategies more often. Meanwhile, Zhang and his associates [35] have expanded the scope of traditional strategy research by integrating learning strategies with elements of self-regulated learning and metacognition. Wang and Bai [36] also noted a positive relationship between self-regulated learning strategies and EFL students’ English learning outcomes in technology using situations.

### 2.2 Koole’s framework for the rational analysis of mobile education

Drawing on both a cognitivist perspective of how learners acquire and understand knowledge [37] and a socio-constructivist perspective that views learning as a dynamic process whereby learners construct knowledge collaboratively with others [38], Koole [39] developed the framework for the rational analysis of mobile education (FRAME), which has been widely used in the literature of educational technology to interpret how learners learn in technology-using environments. Consistent with a socio-constructivist view of learning, FRAME offers a lens to analyze the distinctive characteristics of technology-based language learning as well as the social and personal learning processes involved [40]. According to this framework, the three main aspects involved in a technology-based learning activity, i.e., mobile devices, human learning capacities, and social interaction, depict how learners interact with information through the mediation of technical devices. As the bridge between the learners and the resources, the technical devices need to be of high physical and psychological qualities so that learners can focus on cognitive tasks rather than on the devices. This suggests that the characteristics of technical resources such as input and output capabilities of the technical device all have a significant impact upon students’ use of the resources in learning. On the other hand, for the educational potentials of the technical resources to be best realized and maximized inside or outside the classroom, learner choice, agency, and self-regulation are important factors that will determine the level of learners’ control over their learning. The individual learners need to be equipped with cognitive abilities, prior knowledge, and motivation, and be aware about how they apply their knowledge in new learning situation [41]. Finally, individual learners also need to be capable of communicating, exchanging, and acquiring knowledge with others as social interaction and collaboration are fundamental to learning from a socio- constructivist perspective [42]. As Norton [43] argued, language learning and use are intertwined with social participation and construction of selves which inevitably enhance the opportunities to acquire language use skills and knowledge, and provide learners with feedback, which in turn reinforces students’ learning behaviors.

To summarize, while different interpretations of what SRL is abound, one element of self-regulation that is common to many is viewing SRL as a dynamic process comprising cognitive, motivational and behavioural components which provide learners with the capacity to adjust their actions to achieve particular goals in changing educational settings. Given the above discussion of Koole’s FRAME, we believe that the concept of SRL and the FRAME come together in some interesting ways. Hence, the two sets of theories can be drawn together in this study to analyze Chinese university EFL students’ technology-based self-regulated learning experience.

## 3. This study

Given a lack of consensus in the literature about how to conceptualise and measure technology-based self-regulated learning in an EFL learning context and a pressing need in practice for developing a scale to evaluate students’ engagement in self-regulated use of technology in learning a second or foreign language as discussed above, and answering a call for research to understand the dynamic process of SRL in different learning environments [18, 33], this study aims to address the following two major research questions:

RQ1: What is the factor structure of the technology-based self-regulated English learning strategies scale (TSELSS) for the Chinese university EFL students?

RQ2: How valid and reliable is the TSELSS for the Chinese university EFL students?

### 3.1 Settings and participants

The University of Macau ethics committee has approved this project study. Students' consent to participate in this study was obtained while the project was carried out. A consent form was included in each questionnaire assuring that all responses from the participants would be kept confidential, and that they were also informed that their participation was voluntary and they could withdraw from the study at any time.

Throughout the whole process of this research, two independent samples of Chinese university EFL students participated in this study that consists of three major phases. The first phase involved the generation of an item pool, the second a pilot study aimed at identifying the factor structure of the TSELSS using exploratory factor analysis, and the third an examination of the psychometric properties of the revised TSELSS using confirmatory factor analysis. For the pilot study, a total of 164 (74 males and 90 females) Chinese EFL students were recruited. These participants were first-year and second-year undergraduate students, whose age ranged from 17 to 21 (M = 19.50, SD = 2.28). The participants were from a variety of academic subject backgrounds such as Business, Accounting, and Hotel Management. In the third stage of this study, another sample of 525 (377 males and 148 females) undergraduate students from the same university were recruited to cross-validate the factor structure generated in the earlier phase. This sample of students were also first-year and second-year undergraduate students aged between 17 and 25 (M = 20.50, SD = 7.97), majoring in Finance, Statistics, and Arts.

All the participants were required by the university to take the general College English Course, which is a compulsory course for first-year and second-year students in the university where English teachers and students meet for 3–4.5 hours per week in classrooms. Like all other students, the participants in this study were required by the university to take the nationwide College English Test—Band 4 (i.e., CET-4) before graduation.

### 3.2 Instruments

#### 3.2.1 Technology-based self-regulated English learning strategies for Chinese university EFL students scale (TSELSS)

Drawing on the self-regulated learning theories and the technology-assisted learning perspective in the literature reviewed above, the TSELSS was designed to measure the four major components of technology-based self-regulated English learning strategies: 1) cognitive strategies, 2) metacognitive strategies, 3) social strategies, and 4) motivational regulation strategies.

The items in the TSELSS originated from two major sources: 1) adaptation of items in some existing relevant questionnaires [8, 44–47]; 2) interviews with twenty EFL students about their technology-based English learning experiences. The initial 36 items generated from the literature and interviews with the EFL students were then subjected to judgment of a group of three experts in the field of technology-assisted language learning who were research-active and were also enthusiastic in integrating technology into tertiary-level EFL classroom teaching to examine the face and content validity of the items generated. Specifically, they scrutinized the initial item pools and theoretical rationale, the consistency of construct and the survey questions, as well as the wordings of items. An item was retained only when the majority of the three experts agreed that the item was appropriate for measuring technology-based self-regulated English learning strategies in the Chinese tertiary EFL context. This process resulted in 30 items retained. In the next phase of the TSELSS development, the questionnaire was piloted on a sample of 164 university EFL students for the purpose of identifying the factor structure of the TSELSS through Exploratory Factor Analyses (EFAs). Finally, the factor structure derived from the EFAs was cross-validated on another independent sample of 525 undergraduates through Confirmatory Factor Analyses (CFAs). The results of EFAs and CFAs are reported in the results section below.

### 3.3 Validation measures

#### 3.3.1 The English language self-efficacy questionnaire

Previous studies have already suggested a positive relationship between language learning strategies and students’ language self-efficacy beliefs. Therefore, in this study, the correlations of students’ English language self-efficacy with their technology-based self-regulated English learning strategies were examined to evaluate the concurrent validity of the TSELSS. The English Language Self-Efficacy Questionnaire used in this study contained 16 items that were adapted from Wang and Bai [36], measuring students’ English language self-efficacy in terms of four different domains: speaking, listening, reading, and writing. The participants answered all items on seven-point rating sales (e.g., 1 = not at all true of me, 2 = not true of me, 3 = hardly true of me, 4 = neutral, 5 = almost true of me, 6 = true of me, 7 = very true of me). A Cronbach’s alpha coefficient of 0.97 was found for the total items in the English language self-efficacy questionnaire. Furthermore, the Cronbach’s alpha for the four aspects of English language self-efficacy were: 0.92 for speaking, 0.90 for listening, 0.93 for reading, and 0.93 for writing.

#### 3.3.2 The CET-4

Concurrent validity of the TSELSS was further evaluated by examining the correlation between technology-based self-regulated English learning strategies and students’ English learning outcomes. In this study, CET-4 was used to measure the participants’ English learning outcomes. As an internationally recognized standardized test, CET-4 has been subjected to rigorous validation processes to ensure its high quality as an assessment tool [48, 49]. The CET-4 is administered by the National College English Testing Committee on behalf of the Chinese Ministry of Education [50] to provide an objective evaluation of undergraduate students’ overall English proficiency (State Education Commission, 1986). The test contains four parts: writing (15%), listening comprehension (35%), reading comprehension (35%), and translation (15%) [50].

### 3.4 Data analyses

To answer the two research questions, exploratory and confirmatory factor analyses (i.e., EFAs and CFAs) were conducted by using SPSS 19.0 and M-PLUS 7.4 to identify and confirm the construct validity (factor structure) of the TSELSS. In addition, item analysis, reliability evaluation, and concurrent validity evaluation were also conducted.

## 4. Results

### 4.1 Exploratory factor analyses

Before EFA, Bartlett’s test of sphericity [51] was performed to investigate the factorability of the data, and the Kaiser-Meyer-Olkin (KMO) test [52] was conducted to measure the sampling adequacy. Results showed a significant test statistic for Bartlett’s test of sphericity with the chi-square value of 3069.86 (p < .001), and a KMO value of .92, exceeding the minimum adequacy value of .50 [53]. Then the underlying factor structure of the 30-item TSELSS was examined by EFAs using the principal component extraction method. Five items were excluded from the factor structure due to their low factor loadings or cross-loadings, and a 5-factor model with 25 items was obtained, accounting for 69.62% of the total variance. An internal consistency reliability analysis was then conducted to evaluate the reliability of each subscale, and the Cronbach’s alphas of the five TSELSS factors are: .93 (Motivational regulation strategies), .88 (Goal setting and learning evaluation), .83 (Social strategies), .85 (Technology-based English song and movie learning), and .67 (Technology-based vocabulary learning) respectively (see Table 1), indicating satisfactory levels of internal consistency.

**Table 1:** Results of EFA and reliabilities of the 25-item TSELSS (N = 164).

Factor	Item	Loadings					Eigen value	α
		1	2	3	4	5		
F1. MRS	12. I select and use appropriate technological tools to improve the areas I’m weak in.	**.52**	.37	-.06	-.01	.14	14.12	.93
	15. I use technologies outside the classroom to access authentic materials in English.	**.86**	.20	.01	-.19	-.16		
	17. I search related materials online when I have difficulties in the process of studying English.	**.69**	-.09	.08	.01	.13		
	18. I seek opportunities through technological resources to practice my oral English.	**.76**	.19	-.09	.10	-.05		
	25. I use technologies to help me sustain/enhance interest in learning English.	**.75**	-.16	.06	.26	-.08		
	26. I use technologies to make the English learning task more interesting.	**.84**	.10	-.04	-.09	.07		
	27. I use mobile devices to enhance my willingness to participate in English social events.	**.55**	-.01	.44	-.15	.07		
	29. Sometimes I look through the visual and vivid courseware to arouse my interest in English learning.	**.61**	.01	.19	.18	-.06		
	30. When I feel bored with learning English, I adopt technological resources to decrease the boredom and increase the enjoyment.	**.79**	.13	-.22	.15	.03		
F2. GS	7. I listen to English radio broadcasts (e.g. VOA, BBC) to improve my English proficiency	.16	**.70**	-.07	.14	-.01	2.17	.88
	8. At the beginning of the semester, I set technology-assisted English learning goals.	.04	**.88**	-.19	-.07	.07		
	10. I often monitor my technology-assisted English learning progress.	-.06	**.60**	.01	.38	.13		
	11. I reflect on the effectiveness of using technologies for English learning.	.24	**.59**	.15	-.07	-.05		
	13.I adjust my English learning plans in response to different technology-assisted learning activities.	.38	**.53**	.05	-.06	.03		
F3. SS	21. I seek advice on how to use technologies effectively for English language learning.	.11	.28	**.52**	-.04	-.21	1.56	.83
	22. I. seek opportunities to talk with native English speakers through technological tools.	-.07	.00	**.81**	.16	.02		
	23. When I have problems in English learning, I ask my teacher for help through technological tools.	.13	-.12	**.79**	.11	-.10		
	24. I share my problems with my classmates online so we can solve our problems together.	-.14	-.22	**.90**	.22	.06		
F4. TE	5. I practice saying new expressions in English movies or programs to myself.	-.18	.46	-.04	**.72**	-.06	1.28	.85
	6. I listen to English songs to help me remember words.	-.16	.00	.28	**.77**	.07		
	16. I use technologies (e.g. English movies) to learn more about English and the culture.	.19	-.15	.15	**.67**	.04		
	28. I use technologies to connect English learning with my personal interest (e.g. playing English games, or listening and singing English songs).	.49	-.23	-.06	**.65**	.16		
F5.TV	1. I use lexical apps to help me memorize new words.	-.17	.24	.29	-.13	**.74**	1.06	.67
	2. I use online dictionaries to check English words.	.11	-.11	-.22	.11	**.72**		
	9. I use technologies (e.g. vocabulary apps) to help me persist in my English learning goals.	.03	.42	-.07	.09	**.57**		

Note: MRS: motivational regulation strategies; GS: goal setting and learning evaluation; SS: social strategies; TE: technology-based English song and movie learning; TV: technology-based vocabulary learning.

The first factor contains nine items with factor loadings ranging from .52 to .86, accounting for 47.06% of the variance in the total scale. All the nine items relate to students’ English learning interest enhancement or emotional control with the assistance of technology. Hence, Factor one is labelled as *Motivational Regulation Strategies*.

The second factor is composed of five items with factor loadings ranging from .53 to .88, accounting for 7.22% of the variance in the total scale. This factor is named as *Goal Setting and Learning Evaluation*, which reflects the common characteristics of the five items.

The third factor consists of four items whose factor loadings range from .52 to .90, accounting for 5.20% of the variance in the total scale. The items in Factor three concern students actively seeking for help or looking for learning opportunities by means of technology. Thus factor three was labelled as *Social Strategies*.

The fourth factor contains four items with factor loadings ranging from .65 to .77, accounting for 4.25% of the variance in the total scale. Due to their common characteristics of learning English through songs and movies, this factor is named as *Technology-Based English Song and Movie Learning*.

The fifth factor is composed of three items with factor loadings ranging from .57 to .74, accounting for 3.54% of the variance in the total scale. The items in this factor mainly concern using mobile apps to assist in English vocabulary learning. Hence, Factor five is labelled as *Technology-Based Vocabulary Learning*.

### 4.2 Confirmatory factor analyses

**Fig 1:**
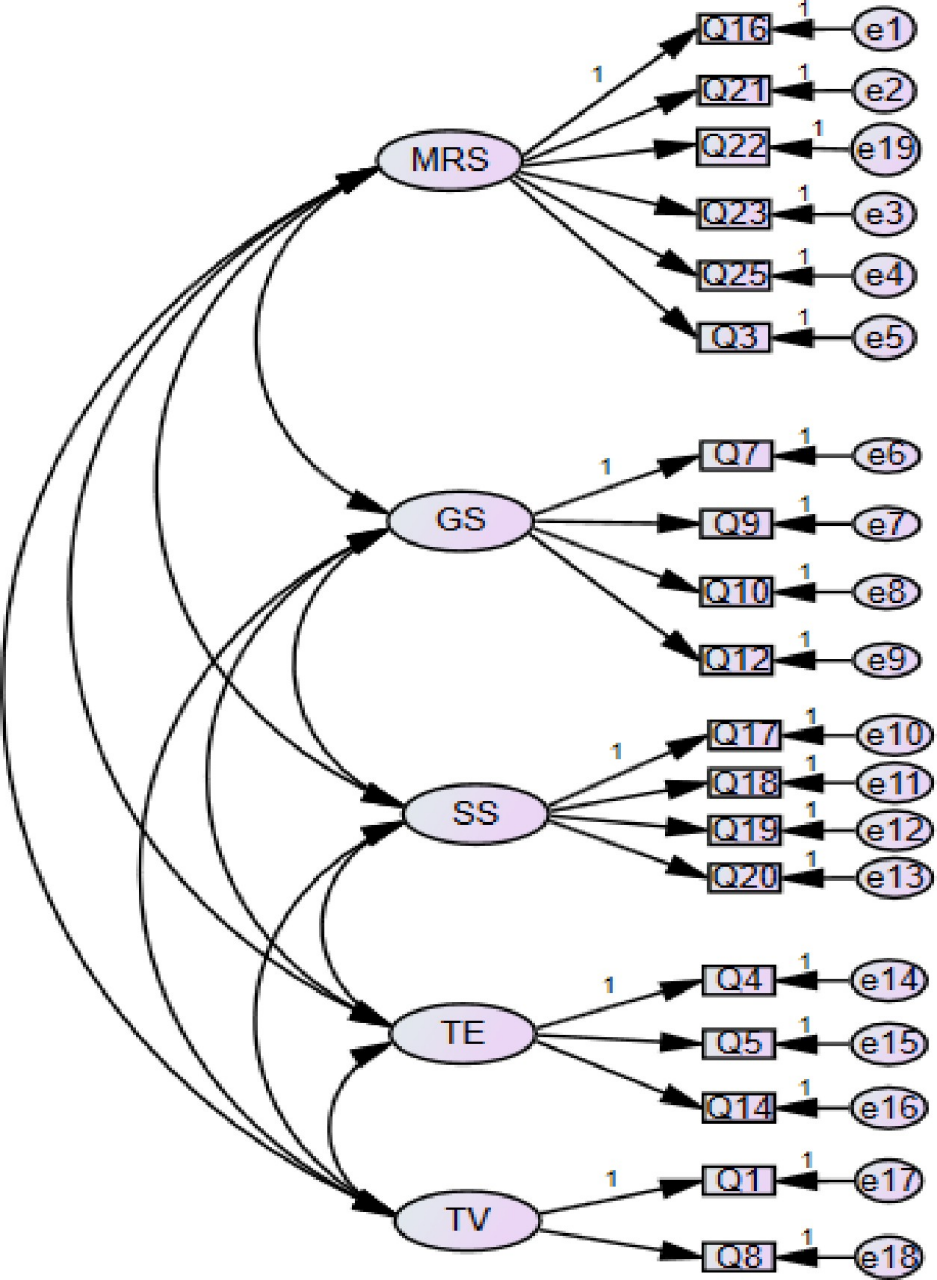
A five-factor model of the 19-item TSELSS. Note: MRS: motivational regulation strategies; GS: goal setting and learning evaluation; SS: social strategies; TE: technology-based English song and movie learning; TV: technology-based vocabulary learning.

To cross-validate the 25-item five-factor structure of the TSELSS generated from EFAs, CFAs were performed in the third phase on the second sample group. The CFA results (X^2^ = 1285.99 (df = 289, *p* < .001); CFI = .88; TLI = .87; SRMR = .05; RMSEA = .08) suggest that the model fit indices were not fully satisfactory. Therefore, we attempted to improve the model fit by addressing the item issues suggested in the modification indices. Six items (i.e., item 2, 6, 11, 13, 15, 24) were removed from the analysis because of the presence of their strong error covariance with other items, and the measurement model was reevaluated. The modified model presented satisfactory model fits with X^2^ = 563.02 (df = 142, p < .001); CFI = .93; TLI = .92; SRMR = .04; RMSEA = .07). Standardized factor loadings for each item ranged from 0.68 to 0.85, with all the factor loadings greater than the benchmark value .50 [21]. The correlations among the five factors were significant (.46-.88), not too close to 1.00 (<0.95) [54]. Fig 1 shows the standardized results for the 5-factor correlated model. In this model, all 19-item parameter estimates were statistically significant (p < .001). Cronbach’s alpha of the subscales ranged from .72 to .89, and Cronbach’s alpha of the whole scale was .94.

### 4.3 A second-order confirmatory factor analysis

A second-order CFA was conducted on the data by loading the five first-order factors onto a second-order factor of technology-based self-regulated strategic English learning. The results suggested an acceptable model fit: X^2^ = 563.94 (df = 145, p < .001); CFI = .93; TLI = .92; SRMR = .04; RMSEA = .07. The standardized regression weights between the second-order factor and the five first-order factors (motivational regulation strategies, goal setting and learning evaluation, social strategies, technology-based English song and movie learning, and technology-based vocabulary learning) were .96, .92, .88, .88, and .67 respectively, and were statistically significant (p < .001), which complied with the recommendation that a large proportion of the second-order loadings should be at least .70 in a second-order construct [55]. Such analysis provides empirical support to considering technology-based self-regulated strategic English learning as a unitary construct with five correlated but distinct sub-types of learning strategies.

### 4.4 Concurrent validity analysis

In the current study, the concurrent validity of TSELSS was evaluated by the Pearson correlation coefficients with students’ English language self-efficacy, and CET-4 scores. The correlation coefficients between the five types of technology-based self-regulated English learning strategies and the overall English language self-efficacy (see Table 2) were statistically significant and positive (rs = .26-.58, ps < .001). In addition, correlations between the five types of technology-based self-regulated English learning strategies and the four dimensions of English language self-efficacy: speaking, listening, reading, and writing, were all significant and positive.

**Table 2:** Correlation between technology-based self-regulated English learning strategies and English language self-efficacy (N = 525).

	Technology-based self-regulated English learning strategies				
	Motivational regulation strategies	Goal setting and learning evaluation	Social strategies	Technology-based English song and movie learning	Technology-based vocabulary learning
English language self-efficacy	.58**	.49**	.56**	.54**	.26**
Speaking	.56**	.46**	.56**	.50**	.21**
Listening	.54**	.46**	.51**	.51**	.23**
Reading	.52**	.46**	.47**	.48**	.27**
Writing	.52**	.45**	.52**	.48**	.26**

** Correlation is significant at the .01 level (2-tailed)

CET-4 was used to measure participants’ English learning outcomes in this study. Table 3 summarized descriptive statistics of students’ technology-based self-regulated English learning strategies and CET-4 scores. The participants’ CET-4 scores (M = 402.80, SD = 54.40) ranged from 200 to 585, indicating that the participants were of different levels of English ability. Results of the Pearson correlation analysis (Table 4) suggest that participants’ English learning outcomes were significantly and positively correlated with four of the five types of technology-based self-regulated English learning strategies (rs = .17-.24, p < .001). Interestingly, only the correlation between CET-4 scores and technology-based vocabulary learning strategies was non-significant.

**Table 3:** Descriptive statistics and reliabilities of the technology-based self-regulated English learning strategies (n = 525).

TSELSS	M	SD	Cronbach’s Alpha
Motivational regulation strategies	4.25	1.30	0.89
Goal-setting and learning evaluation	4.03	1.29	0.79
Social strategies	3.73	1.41	0.86
Technology-based English song and movie learning	4.11	1.37	0.78
Technology-based vocabulary learning	4.89	1.43	0.72
CET-4 score	402.80	54.40	

**Table 4:** Correlations between technology-based self-regulated English learning strategies and English learning outcomes (N = 525).

	Technology-based self-regulated English learning strategies				
	Motivational regulation strategies	Goal setting and learning evaluation	Social strategies	Technology-based English song and movie learning	Technology-based vocabulary learning
CET4	.24**	.17**	.20**	.24**	.08

** Correlation is significant at the .01 level (2-tailed)

## 5. Discussion

The main goal of the present research was to develop and validate the TSELSS, a multidimensional scale for the evaluation of technology-based self-regulated English learning strategies in Chinese university EFL students. By means of exploratory and confirmatory factor analyses, five factors were determined: *motivational regulation strategies*, *goal setting and learning evaluation*, *social strategies*, *technology-based English song and movie learning*, and *technology-based vocabulary learning*. Given the remarkable expansion of technology‐mediated language teaching and learning over the past decades, knowledge of what technology-based self-regulated English learning strategies students prefer and what strategies were possibly omitted in the previous research will be useful in providing support and designing student training programs to enhance their use of technology in English language improvement.

The CFA results supported the internal construct validity of the TSELSS on both the dimension level and the item level. The absolute and comparative fit indices suggested that the CFA model fitted our sample data well. Moreover, all the items significantly loaded on their corresponding subscales, and these subscales all loaded significantly on a common technology-based self-regulated strategic English learning factor as a single underlying construct, providing evidence that the scale is multidimensional, being composed of the different five dimensions. These results therefore confirm that the TSELSS has good internal construct validity among Chinese tertiary EFL learners, and that our conceptualization of technology-based self-regulated strategic English learning as a unitary construct is coherent both conceptually and from the student perspective. Consequently, the scores of the 19 TSELSS items can be collectively summed to reflect an EFL learner’s overall technology-based self-regulated English learning strategy use, or calculated separately to represent the use of each particular type of technology-based self-regulated English learning strategies in the Chinese tertiary EFL context. In addition, the wide distribution of the participants’ CET-4 scores in the current study indicated that the TSELSS can likely be applicable for EFL learners of a variety of language levels.

Recent research in educational psychology points to the importance of regulation of motivation in self-regulated learning. Self-regulated students have been found to be able to control their affect and emotions through the use of various coping strategies that help them deal with negative affect such as anxiety and boredom [18]. The existing instruments, for example, the OSLQ, developed by Barnard and her colleagues [8] for measuring online self-regulation, failed to cover the aspect of motivational or affective regulation. Similarly, the widely used instrument measuring self-regulated learning, MSLQ, does not include scales that assess any strategies to control motivation or affect, given the importance of motivational strategies in self-regulated learning [24]. In extending Pintrich et al.’s work, we succeeded in verifying motivational regulation as a dimension of technology-based self-regulated strategic English learning. This result provides empirical evidence that Chinese university EFL students are able to differentiate between motivational regulation and other forms of self-regulated learning strategies such as metacognitive, cognitive and social strategies.

In the present study, the concurrent validity of TSELSS was examined by exploring the correlations between five types of technology-based self-regulated English learning strategies and English language self-efficacy, as well as English learning outcomes. The five types of technology-based self-regulated English learning strategies were found to be positively related to both the participants’ overall English language self-efficacy and its four domains (i.e., speaking, listening, reading, and writing). These results were in line with previous research [34] that suggests that high self-efficacy learners tend to be more cognitively, metacognitively, and motivationally engaged in learning. In addition, the current study revealed significantly positive correlations between four of the five types of technology-based self-regulated English learning strategies and the participants’ English learning outcomes, echoing the observation in educational psychology that self-regulated learning strategies positively influence students’ academic learning achievements.

Note that in this study, students’ mean scores in five types of technology-based self-regulated English learning strategies ranged from 3.73 to 4.89 on a 7-point Likert scale (see Table 3). Technology-based vocabulary learning strategies were reported to be the most frequently used strategies, followed by motivational regulation strategies, technology-based English song and movie learning, goal-setting and learning evaluation, and social strategies. One possible reason for a seemingly high level of use of technology-based vocabulary learning strategies might be that participants in our research were under the pressure of coping with CET-4, and memorizing English vocabulary words was widely assumed to be the most important part of the test preparation effort in China. Nevertheless, these technology-based vocabulary learning strategies were found to be weakly correlated with students’ English language self-efficacy and nonsignificantly correlated with their English learning outcomes. What might account for this weak impact of technology-based vocabulary learning strategies is that the vocabulary learning strategies students used heavily relied on memorization, a surface learning strategy which in fact may not lead to long-term retention of words [56, 57]. In our interviews with some EFL students in the questionnaire development stage, some students commented that their English learning after class occurred largely in the form of vocabulary words memorization as they tended to believe that so long as they developed a large vocabulary, they could automatically read and write in English. Research on second language acquisition, however, suggests that the best ways of learning English vocabulary are through reading or through putting new words into writing and speaking. It is thus likely that ineffective vocabulary learning such as words memorization here is likely to disadvantage EFL students in the National College English Test (i.e., CET-4) which focuses on testing students’ English reading and writing abilities. This result suggests a pressing need for teachers providing in-class guidance to help students to adopt more effective ways of English vocabulary learning.

In the dimension of motivational regulation strategies of the TSELSS, a mean score of 4.25 suggests this type of strategy is generally frequently used, which is encouraging and indicates that Chinese tertiary EFL students were relatively confident in maintaining their motivation and controlling their affect and emotions through the use of various coping strategies that help them deal with negative affect such as boredom. Significantly, motivational regulation strategies were found to be most powerfully positively correlated with both English language self-efficacy and English learning outcomes than any other types of technology-based self-regulated English learning strategies identified in this study. It is thus likely that students’ regulation of motivation for learning may result in students being more committed to the tasks they are engaged in, which in turn likely contributes to higher English language self-efficacy and better English learning outcomes. An important implication of our finding is thus that current technology interventions aimed at promoting use of educational technology among students in higher education institutions need to shift from a focus on training of students’ technological skills *as outlined in TESOL Technology Standards* [58] to paying more attention to development, maintenance and regulation of students’ motivation in use of technology for foreign or second language learning.

At the same time, our data showed that technology-based social English learning strategies were least frequently used among the participants. This result is consistent with the finding from Lai and Gu [9] that foreigner language learners in their study were skeptical about using technology to create social learning opportunities and support beyond their immediate social network, and that they generally expressed discomfort in interacting with native speakers. This may be particularly the case with EFL learners in China where students like more to interact and seek help from peer classmates due to their generally low level of English speaking proficiency.

## 6. Conclusion

Given a general lack of multidimensional conceptualisation or instrumentation for measuring technology-based self-regulated English learning strategies, this study has made a theoretical contribution by conceptualising Chinese university EFL students’ technology-based self-regulated English learning strategies as having five discrete dimensions and by empirically testing this theoretical assumption. Importantly, the present findings are probably the first that consistently showed that motivational regulation strategies demonstrated the most powerful associations with learners’ English language self-efficacy and English learning outcomes.

Pedagogically, knowing students’ preference for technology-based self-regulated English learning strategy use is helpful for teachers to initiate appropriate remedial actions for improvement if students are found to be less users of any self-regulated English learning strategies. Furthermore, a knowledge of what types of strategies best contribute to students’ English learning outcomes enables teachers to focus on promotion of those strategies needed by certain categories of students. The TSELSS can also be used by researchers to examine the relationship between use of technology-based English learning strategies and some other teaching and learning factors. For example, given the current movement of promoting self-regulated learning as a worthy instructional goal, researchers can adopt the TSELSS to explore the influence of different kinds of teaching approaches on students’ use of technology-based self-regulated learning strategies.

Although this study provided evidence that the TSELSS constitutes a reliable and valid instrument for measuring relevant dimensions of technology-based self-regulated English learning in tertiary EFL students, there are limitations that need to be acknowledged and addressed by future research. First, participants in our study were at the same instructional level and from the same university, which may limit the generalization of the instrument to other populations. Therefore, future research is recommended to employ samples from different universities in different regions to see if the response patterns are the same and if the intercepts and loadings of factors are the same across populations. This will allow us to explore context-dependent nature of such strategies through comparative research. Second, an additional limitation of the this study is that due to the cross-sectional nature of the data, the results only indicate associations between self-regulated English learning strategy use and other variables such as English language self-efficacy and English learning outcomes, future studies with a longitudinal or experimental design are suggested to establish causal claims.

## Supporting information

S1 Appendix Chinese version of the TSELSS. (DOCX)

S1 Data (SAV)
