# Supplementary material for: Profiling Chinese EFL students’ technology-based self-regulated English learning strategies
Source: PLoS One. 2020 Oct 29;15(10):e0240094. doi: 10.1371/journal.pone.0240094 (PMC7595288; doi:10.1371/journal.pone.0240094)
Supplement: S1 Appendix — (DOCX) [file pone.0240094.s001.docx]

**S1 Appendix**. Chinese version of the TSELSS

1. 我使用词汇软件帮助我记单词。
2. 当我对学英语感到无聊时，我利用技术辅助学习来减少无聊感、提升兴趣。
3. 我练习说英语电影或英语节目中所学到的新的表达方式。
4. 我听英文歌帮助自己记单词。
5. 新学期开始时，我设定相应的技术辅助英语学习目标。
6. 我使用相关的技术工具（比如：词汇软件）帮助我坚持英语学习目标。
7. 我经常监测自己在技术辅助英语学习方面的进展情况。
8. 我对技术辅助英语学习的效率进行反思。
9. 针对不同的技术辅助英语学习任务，我会相应的调整学习计划。
10. 我通过网络资源（比如：英文电影）学习有关英语及其文化方面的知识。
11. 我用技术资源搜索可以练习英语口语的机会。
12. 我向别人寻求关于如何有效使用技术辅助英语学习的意见。
13. 我通过技术资源寻找机会与英语为母语的人交流。
14. 当我英语学习遇到困难时，我用手机等技术工具向老师寻求帮助。
15. 我在网上与同学分享我的问题，这样我们可以一起想办法解决。
16. 我使用多媒体英语学习帮助自己保持或提升英语学习兴趣。
17. 我运用英语学习软件或网站使学习任务更加有趣.
18. 我用手机帮助自己提升参加英语社交活动的意愿。

我看生动有趣的学习课件来提升自己对英语的学习兴趣。
